# Single-Isocenter, Multiple-Target Abdominal Cone-Beam Computed Tomography (CBCT)-Guided Online Adaptive Stereotactic Body Radiotherapy (SBRT)

**DOI:** 10.7759/cureus.68904

**Published:** 2024-09-07

**Authors:** Chipo Raranje, Thomas R Mazur, Allen Mo, Eric Laugeman

**Affiliations:** 1 Radiation Oncology, Washington University School of Medicine, St. Louis, USA

**Keywords:** cone-beam computed tomography, motion management, oligometastases, online adaptation, single-isocenter and multiple-target

## Abstract

Stereotactic body radiotherapy (SBRT) is increasingly being prescribed for treating patients with multiple metastases, especially in the setting of oligometastatic disease. Treating multiple targets presents unique challenges in radiotherapy planning and delivery, including practical considerations relating to treatment time, resource allocation, and treatment planning complexity. Treating targets in a common isocenter reduces the time required for treatment and simplifies planning, but historically, it has often not been feasible due to inter- and intra-fractional variation in relative target positions. With online adaptation, individual targets can be re-contoured on each treatment fraction to obviate inter-fractional variation, and with appropriate margin selection intra-fractional motion can be managed.

In this case report, we describe single-isocenter, multiple-target treatment via online adaptation of a 93-year-old man with a history of metastatic hepatocellular carcinoma. He initially presented with a 9.1 cm liver mass, suspicious lung lesions, and an enlarged porta hepatis lymph node, which were biopsy proven to be hepatocellular carcinoma. Following 18 months of systemic immunotherapy, he demonstrated a favorable response, including a reduction in primary liver mass to 5.1 cm and resolution of pulmonary lesions; however, recent serial imaging demonstrated oligoprogression of two peripancreatic lymph node conglomerates that were biopsy proven to be poorly differentiated carcinoma. The patient was offered adaptive SBRT to a dose of 35-40 Gy in five fractions as a consolidative approach for treating both the primary liver mass and oligoprogressive lymph nodes. He tolerated treatment without any grade 2 or higher acute toxicity and had stable disease on three-month post-treatment imaging.

By leveraging online adaptation, especially for the daily re-definition of target volumes, we were able to treat three targets in the abdomen accurately in a common isocenter. Treating in this manner vastly shortened and simplified the patient's radiation course. Quantitative evaluation of re-contoured targets and post-treatment imaging highlighted the value of online adaption with careful margin specification and alignment instructions.

## Introduction

Multiple studies have recently demonstrated the safety and efficacy of treating patients with stereotactic body radiation therapy (SBRT) to multiple metastases [1,2]. Largely due to promising results in these studies, SBRT has become increasingly common in the treatment of patients with oligometastases [3]. For patients with just a few sites of metastasis, SBRT, often combined with immunotherapy, has enabled prolonged progression-free and overall survival and even motivated treatment of metastatic disease with curative intent [4-8]. As SBRT expands in application to oligometastatic disease, technical complexity in treatment planning and delivery continues to increase. When treating many metastases in a common course, practical challenges include scheduling and delivering treatment in manageable time, both in terms of patient comfort on the treatment table and allocation of clinical resources. Treatment planning can be challenging when metastases are sufficiently close, and the dose between and adjacent to targets is difficult to control. Delivered doses both to targets and adjacent organs at risk (OARs), in turn, can be highly sensitive to residual error in patient setup. 

Linac-based intracranial stereotactic radiosurgery (SRS) highlights the challenges in treating multiple targets in proximity to each other [9-11]. In single-isocenter, multiple-target (SIMT) technique targets can be simultaneously treated in a common plan; however, accuracy for targets away from the isocenter is highly sensitive to residual rotational error [12]. For these reasons, margin selection is an important consideration for SIMT, even when treating brain metastases where relative target positioning is generally not susceptible to inter-fractional variation [13]. While principles applied to SIMT for SRS can be translated to treating extracranial disease, thoracic and gastrointestinal (GI) targets present unique challenges due to inter- and intra-fractional motion. 

Given that inter-fractional variation can often be unpredictable, extra-cranial multi-target courses generally favor treating each target in its own isocenter to ensure the accuracy of each treatment. When requiring motion management such as respiratory gating, treatment time can become prohibitively long and possibly require targets to be treated on separate days. Moreover, when targets are sufficiently close, the plan design for each isocenter must account for the position of the untreated targets to ensure that spill on composite dose is well-controlled [14,15]. With online adaptation, these challenges can be largely circumvented by re-contouring target volumes on imaging-of-the-day. Systematic intra-fractional variation, i.e., a drift of one target relative to another mid-treatment, can be managed by tailoring setup margins to provide alignment flexibility for a target in a more forgiving location relative to surrounding OARs [16]. Rule-based auxiliary structures can then be derived each day from target volumes to control dose to OARs and tissues between targets. 

In this case study, we describe the application of online adaptation for treating three targets in a single isocenter via SBRT. We specifically describe margin selection and rule definitions for streamlined online re-planning. We also summarize inter- and intra-fractional variation observed during this course to assess the suitability of our planning and treatment strategy. Given that online adaptation is often applied toward iso-toxic irradiation of target surrounded by dose-limiting OARs, we present this case as a unique application of ART for accelerating treatment time and achieving the highest-quality plan dose for a complicated assortment of oligometastases.

## Case presentation

Clinical presentation

This is the case of a 93-year-old male with a history of chronic lymphocyte leukemia on active surveillance and metastatic hepatocellular carcinoma who initially presented with a 9.1 cm liver mass, suspicious lung lesions, and an enlarged porta hepatis lymph node, which was biopsy-proven to be hepatocellular carcinoma. He was started on ipilimumab and nivolumab for over 18 months with favorable partial response, including reduction in primary liver mass to 5.1 cm and resolution of pulmonary lesions. Recent serial imaging demonstrated oligoprogression of two peripancreatic lymph node conglomerates that were biopsy proven to be poorly differentiated carcinoma presumed to be hepatocellular origin. 

Given progressive disease, the patient was offered adaptive SBRT as a consolidative approach for treating both the primary liver mass and oligoprogressive lymph nodes. The prescribed dose was 40 Gy in five fractions to the primary liver lesion and 35 Gy in five fractions to the two lymph nodes. The patient tolerated treatment without any reported grade 2 or higher acute toxicity by Common Terminology Criteria for Adverse Events. He has continued on ipilimumab and nivolumab after radiation treatment with stable disease on post-treatment CT imaging at three months.

Treatment planning and delivery

Simulation and Planning

In the interest of reducing both the days required for completing the course and the time each day on the treatment table, a single plan was prepared to treat all targets in a common isocenter using the Varian Ethos online adaptive platform (Varian Medical Systems, Palo Alto, CA). Dose constraints for luminal gastrointestinal (GI) OARs, including stomach, duodenum, small bowel, and large bowel, were D0.5cc < 33 Gy. The patient was simulated in a patient-specific mold with both arms positioned above his head. End-exhale breath-hold CT and retrospective phase-binned four-dimensional (4D)CT images were acquired, and treatment planning was performed on an average of exhale phases from the 4DCT reconstruction. 

**Figure 1:**
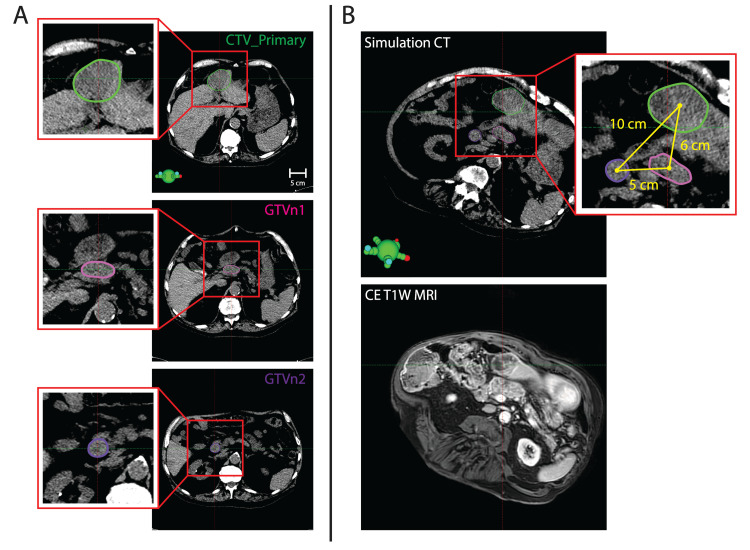
Target summary A) Overview of target volumes, including clinical target volume (CTV)_Primary (liver) and gross tumor volume (GTV)n1 and GTVn2 (peripancreatic lymph node conglomerates), as shown on simulation CT.  B) Summary of distances between targets on simulation CT imaging and diagnostic contrast-enhanced T1weighted (T1W) MRI.  Insets zoom in around the target volumes as indicated by red boxes and lines.

As illustrated in Figure 1, the primary liver gross tumor volume (GTVp) and two lymph nodes (GTVn1 and GTVn2) were contoured separately and were localized directly on simulation CT imaging with guidance from co-registered T1W MRI. A physician-drawn clinical target volume (CTV_Primary) was specified based on the GTVp. The planning target volume (PTV) margin from CTVp was an isotropic 5 mm. PTV margin from GTVn1 and GTVn2 was an isotropic 7 mm in an effort to provide a buffer for intra-fractional motion between the targets that might require fine adjustments to patient alignment. Post-adaptation imaging focused alignment on the CTV_Primary, and then verified that GTVn1 and GTVn2 remained confined within their associated PTVs.

The treatment plan was prepared using the volumetric modulated arc technique (VMAT) using three full arcs with unique collimator angles selected in an effort to enable dose carving between targets. Isocenter was positioned near the center of mass of the combined PTVs. Structure rules were prepared for daily adaptation defined PTV_Opt structures by cropping each PTV from the union of gastrointestinal (GI) OARs. PTV_Opt structures were cropped 5 mm from OARs for the primary target and 3 mm for the nodal volumes.

Online Adaptation and Delivery

Cone-beam computed tomography (CBCT) imaging was acquired for each fraction at end-exhalation breath-hold as confirmed by an optical surface monitoring system. A region of interest (ROI) was manually specified on the abdomen for optical tracking. Upon updating the treatment plan via adaptation and for any subsequent couch shift, a reference surface was captured by the cameras to re-define the patient’s end-exhalation position. Beam holds during imaging and treatment were performed manually by confirming that the tracked ROI matched its average reference position within a window of ±2 mm.

**Figure 2:**
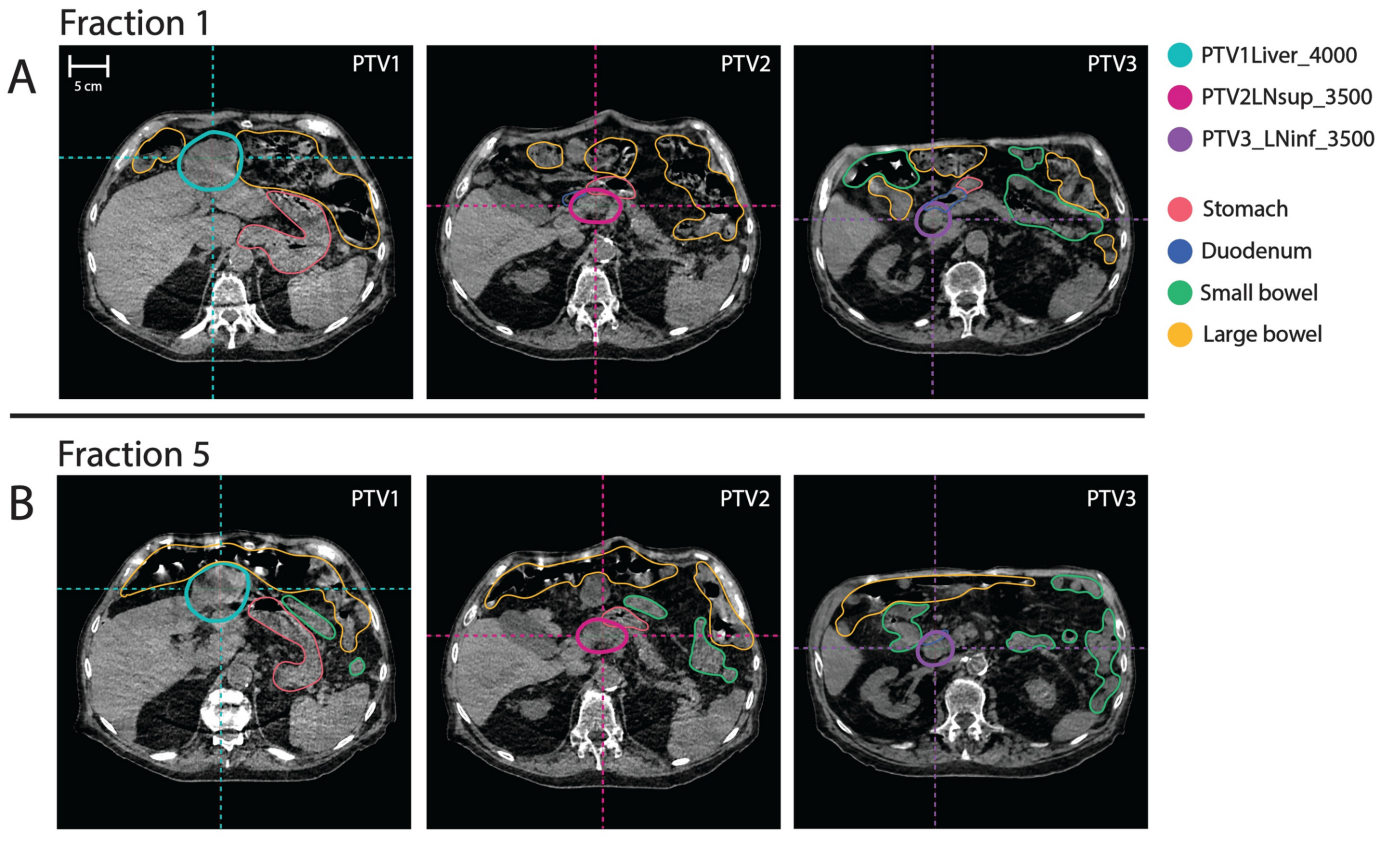
Daily CBCTs for online adaptation Examples of target definitions on fractions 1 and 5 relative to adjacent gastrointestinal (GI) organs at risk (OARs) on cone beam computed tomography (CBCTs) acquired for those fractions.

CTV and GTVs were rigidly propagated independently onto the planning CBCT for each fraction. On four of five fractions, at least one propagated target was manually edited by a covering radiation oncologist. Normal tissue structures were manually edited as visualized on CBCT inside of a 3 cm expansion of the combined PTVs. As exemplified in Figure 2, PTVs and associated structures were then prepared according to specified rules. An initial plan was re-calculated for each fraction on the scan-of-the-day (scheduled plan) and the plan was simultaneously re-optimized on this scan to maximize target coverage while ensuring priority 1 GI OAR constraints were met (adapted plan). 

Planning criteria were compared between scheduled and adapted plans. The adapted plan was selected for treatment if any priority 1 constraints were unmet on the scheduled plan or if target coverage on any PTV improved by more than 5%. Table 1 summarizes target coverages on scheduled and adapted plans for all fractions. The adapted plan was selected for all fractions due to unmet priority 1 large bowel, duodenum and/or stomach constraints. Table 2 provides fraction-by-fraction D0.5cc values for the stomach, duodenum, and large bowel for comparison.

**Table 1:** Fraction-by-fraction target coverage Comparison of target coverages (D95%) on scheduled and adapted plans for each fraction. PTV: Planned target volume

	Scheduled (Gy)			Adapted (Gy)		
Fraction	PTV1	PTV2	PTV3	PTV1	PTV2	PTV3
1	5.4	4.5	5.0	6.6	5.7	5.3
2	6.9	5.2	5.1	6.6	5.5	5.3
3	7.1	5.4	5.2	6.5	5.7	5.3
4	7.2	4.7	5.0	6.8	5.4	5.2
5	6.5	4.9	4.8	6.0	5.6	5.2

**Table 2:** Comparison of per fraction D0.5cc (Gy) values between scheduled and adapted plans. Overview of D0.5cc (Gy) to stomach, duodenum and large bowel for each treatment fraction on both scheduled and adapted plans.  Bolded values denote unmet constraints on scheduled plans, where the planning constraint is D0.5cc < 33 Gy.

	Per Fraction D0.5cc (Gy)										
		Fraction 1		Fraction 2		Fraction 3		Fraction 4		Fraction 5	
	Pre-plan	Scheduled	Adapted	Scheduled	Adapted	Scheduled	Adapted	Scheduled	Adapted	Scheduled	Adapted
Stomach	6.3	6.2	6.2	7.5	6.3	6.9	6.4	7.1	6.3	6.3	6.3
Duodenum	6.0	7.3	6.3	6.2	6.2	6.1	6.2	6.1	6.0	5.9	6.2
Large Bowel	6.0	7.7	6.3	8.5	6.3	8.5	6.2	8.5	6.1	8.6	6.5

In several fractions, PTV coverage improved with adaptation by greater than 10% because of target migration relative to initial positioning at simulation. Table 3 highlights fraction-by-fraction variation in individual target positions by calculating Dice similarity coefficients (DSCs) between adapted PTV contours and associated PTVs defined on simulation CT. DSCs highlight that on fraction 5, the target position deviated greatest from simulation, which in turn contributed to the largest observed differences in coverage between scheduled and adapted plans.

**Table 3:** Dice similarity coefficients Dice similarity coefficients calculated between 1) planned target volumes (PTVs) rigidly propagated to daily cone beam computed tomography (CBCT) imaging as a summed contour and 2) PTVs propagated independently to daily imaging and manually edited as required for localization.

Fraction	Dice Similarity Coefficient		
	PTV1	PTV2	PTV3
1	0.86	0.85	0.80
2	0.94	0.85	0.82
3	0.94	0.89	0.87
4	0.89	0.75	0.75
5	0.67	0.77	0.58
Average	0.86	0.82	0.76

**Figure 3:**
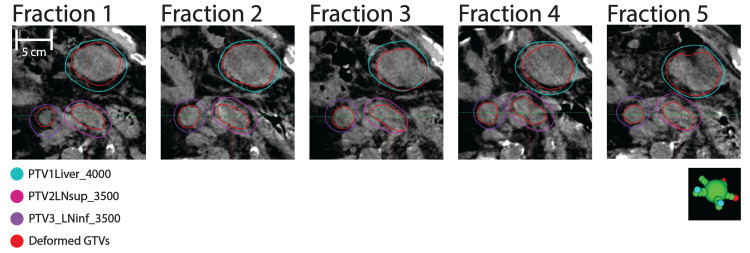
Post-treatment CBCT imaging Comparison of clinical target volume (CTV) and gross tumor volume (GTV) contours (red) deformably mapped onto post-treatment cone beam computed tomography (CBCT) imaging for each fraction relative to PTV contours rigidly mapped based on couch position.

CBCT was re-acquired whenever the exhale position did not match its reference position defined by optical tracking. Additionally, mid- and post-treatment CBCTs were acquired to confirm target alignment relative to optical monitoring. Figure 3 shows post-treatment CBCTs acquired on each fraction, with PTV contours overlaid and CTV/GTV contours deformably mapped from the CBCT acquired for adaptation. No couch shifts are specified on these final acquisitions.

Table 4 summarizes margins required for CTV and GTV contours defined at adaptation to confine the deformed targets propagated onto the post-treatment acquisitions. For most fractions and targets, this margin (rounded to the nearest mm) did not exceed the planning margin. On two fractions, a small volume of deformed GTV extended beyond the PTV margin for either one or two targets. By retrospectively fine-tuning the registration defined on mid-treatment imaging to focus on CTV_Primary alignment per intended instructions, residual error was reduced for these two fractions.

**Table 4:** Post-treatment alignment Summary of margin that must be applied to daily clinical target volume (CTV) and gross tumor volume (GTV) contours to encapsulate deformably-mapped CTV and GTVs on post-treatment cone beam computed tomography (CBCT) acquisitions on each fraction.  Asterisks indicate results obtained with retrospectively updated registrations applied between initial and post-treatment CBCTs that prioritize the accuracy of CTV_Primary.

	CTV_Primary		GTVn1		GTVn2	
Fraction	Margin (mm)	Confined?	Margin (mm)	Confined?	Margin (mm)	Confined?
1	5	No	3	Yes	5	Yes
1*	5	Yes	2	Yes	4	Yes
2	3	Yes	3	Yes	0	Yes
3	6	No	7	No	5	Yes
3*	2	Yes	6	No	3	Yes
4	3	Yes	2	Yes	2	Yes
5	3	Yes	6	Yes	4	Yes

## Discussion

In this case report, we describe the application of CBCT-guided adaptive radiotherapy for treating multiple abdominal targets in a single-isocenter plan. This case illustrates how online adaptation can be leveraged to accurately, efficiently, and safely treat multiple targets adjacent to GI OARs. For all fractions, treating with the scheduled plan would have resulted in at least one OAR violating a highest-priority dose constraint. In addition, adaptation allowed for improved localization accuracy, and also, in most fractions, greater target coverage. Other studies generally focus on planning techniques for mitigating challenges relating to multiple-target, single-isocenter treatment without adaptation [17-20]. Few reports describe the simultaneous treatment of multiple targets in a common isocenter with adaptation, and these studies treat in the lung where synchronous motion may be expected and intra-fractional motion of one target relative to another may be less likely [21]. 

On two fractions, as shown in Table 4, deformably mapping CTV and GTV onto a post-treatment CBCT indicated that targets may extend beyond the PTV margin by up to 1 mm. The fractional volume of the deformed target beyond PTV in these instances was <0.1 cm^3^. On retrospective review of one of these fractions (fraction 1), no mid-treatment shifts were applied, given that CTV and GTVs were confined in PTVs. However, a shift could have been applied to minimize residual error in CTV alignment based on intended targeting prioritization. As shown in Table 4 (indicated by the asterisk), targets would have been confined in PTV post-treatment had this shift been applied. On the other fraction (fraction 3), mid-treatment shifts were applied, but similarly, CTV alignment was not prioritized. Had alignment focused on maximizing CTV accuracy, post-treatment target confinement would have improved. These results highlight the importance of providing simple alignment instructions for the treating team and adhering to those instructions.

The median (range) treatment time over all fractions was 106 minutes (23 minutes), with the first and third fractions being the longest (109 minutes). The minimum allocated time for an SBRT treatment fraction with breath-hold technique on Ethos at our institution is 50 minutes. Treating these targets consecutively on the same day likely would have required at least two hours to be reserved for each treatment fraction. Moreover, more imaging would be required to shift isocenter-to-isocenter and update patient alignment. More breath-holds likely would be required by the patient because the cumulative monitor units on three plans would likely exceed the total number in a single plan. Multiple isocenter plans can be staggered over additional days at the expense of clinical resources and patient visits, which can be burdensome, especially for patients commuting from a distance.

**Figure 4:**
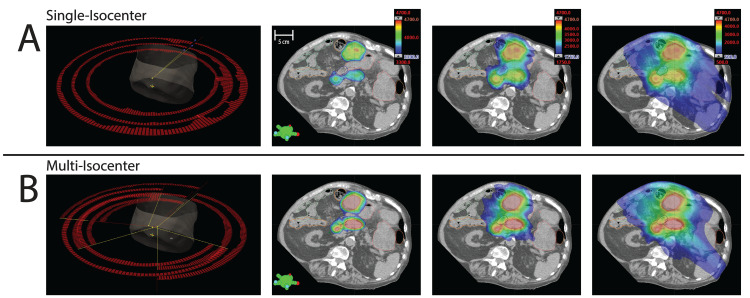
Plan approach comparison Comparison of arc design and total dose for the A) initial single-isocenter plan (upper) and a B) candidate three-isocenter series of plans.

For targets on overlapping axial planes in proximity to each other, controlling composite dose can be challenging especially in spaces between targets. Figure 4 compares the dose distribution for the native single-isocenter plan to a candidate three-isocenter plan sum. In preparing plans per target, arcs for each plan are tailored in an effort to limit cumulative dose spill between targets, especially in adjacent OARs. Optimization objectives are included in each plan to limit dose spills into the un-targeted volumes, which can lead to high modulation and atypical dose spills. Other objectives are also included to ensure OAR constraints are met on a planned sum, and these often require ad hoc and hand-crafted structures that are difficult to standardize and likely not amenable to online adaptation. In contrast to the three-arc scheduled plan requiring approximately 5000 monitor units (MUs), the three plans combined in Figure 4 for a multi-isocenter approach include eight arcs total and more than 6000 MUs.

On CT imaging two months post-treatment, all targets demonstrated reduced size. The patient tolerated treatment without any grade two or higher toxicities during his post-treatment follow-up. He did experience grade 1 fatigue and nausea immediately post-treatment but did not have grade two or higher treatment-related toxicities. He had an episode of vasovagal syncope resulting in a fall without loss of consciousness two months after treatment, where he was discharged from the emergency department after evaluation. He has had a normal serum AFP since diagnosis of his hepatocellular carcinoma, and a comprehensive metabolic panel, including liver function, remained stable after treatment, with a stable albumin-bilirubin (ALBI) grade of 2.

As online adaptation increases in popularity in our clinic, finding ways to reduce treatment course duration and patient time-on-table is of paramount importance to ensure throughput and promptness in care. Multiple research directions strive to reduce adaptation time, especially key steps such as OAR re-contouring and plan re-optimization. Multi-target treatments have historically challenged our clinical efficiency because individual steps in the treatment process must be repeated for each isocenter. As demonstrated in this report, online adaptation can accelerate these treatments with careful considerations of localization and motion management. Even in instances where the target dose may not be limited by adjacent OARs, online adaptation can support improved localization accuracy and precision by independent target alignment and re-contouring.

## Conclusions

By leveraging online adaptation, especially for daily re-definition of target volumes, we were able to treat three targets accurately in the abdomen in a common isocenter. Treating in this manner vastly shortened and simplified the patient's radiation course. Quantitative evaluation of re-contoured targets and post-treatment imaging highlighted the value of online adaption with careful margin specification and alignment instructions.
